# Comparative Transcriptomics Reveals the Molecular Basis for Inducer-Dependent Efficiency in Gastrodin Propionylation by *Aspergillus oryzae* Whole-Cell Biocatalyst

**DOI:** 10.3390/biom15121695

**Published:** 2025-12-04

**Authors:** Desheng Wu, Maohua Ma, Xiaohan Liu, Xiaofeng Li, Guanglei Zhao

**Affiliations:** 1 School of Light Industry, Liming Vocational University, Tonggang West Street 298, Quanzhou 362000, China; deshengwu@lmu.edu.cn; 2 School of Food Science and Engineering, South China University of Technology, Wushan Road 381, Guangzhou 510640, China; 3 State Key Laboratory of Pulp and Paper Engineering, South China University of Technology, Guangzhou 510641, China

**Keywords:** *Aspergillus oryzae*, biocatalysis, transcriptome, lipase, gastrodin

## Abstract

Propionylated derivatives of gastrodin are valuable due to their enhanced lipophilicity and bioavailability. This study investigated the molecular basis for the differential catalytic efficiency of *Aspergillus oryzae* whole cells in gastrodin propionylation. A high conversion rate of 96.84% was achieved with soybean oil induction, compared to only 8.23% under glucose induction. Comparative transcriptomic analysis identified 20,342 differentially expressed genes (DEGs), which were significantly enriched in lipid metabolism and signal transduction pathways. From 26 upregulated lipase-related DEGs, a candidate triacylglycerol lipase gene (CL24.Contig40_All) was prioritized. Homology modeling and molecular docking supported its potential role by demonstrating that the encoded enzyme possesses a typical α/β hydrolase fold with a catalytic triad and favorable binding with both gastrodin and vinyl propionate. These findings indicate that soybean oil may enhance lipase expression by activating lipid metabolic and phosphatidylinositol signaling pathways, providing crucial transcriptional-level insights and genetic targets for the rational design of efficient whole-cell biocatalysts.

## 1. Introduction

Gastrodin, a natural phenolic glycoside derived from the traditional medicinal plant *Gastrodia elata*, has been widely documented to possess various pharmacological activities, including neuroprotection, sedation, anticonvulsant effects, and memory enhancement, with high safety and few reported side effects [1,2]. This compound has a long history of use in China and is widely employed as a functional ingredient in food processing, recognized as a medicinal and edible substance [3]. However, its polar glycoside structure results in low lipophilicity and bioavailability, limiting broader clinical applications [4,5]. To improve bioavailability, structural modification, particularly acylation, has become an important research direction for synthesizing more liposoluble derivatives [6,7].

Driven by the principles of green chemistry, enzymatic acylation has emerged as a promising strategy to replace traditional chemical catalysis, owing to its mild conditions, high regio- and stereoselectivity, and environmental friendliness [8]. Lipases, as key enzymes catalyzing transesterification or esterification reactions, have been extensively applied [9]. However, the use of isolated and purified enzymes faces challenges such as high cost and poor operational stability [10]. To address these limitations, whole-cell biocatalysis has been developed [11]. This technique utilizes intact microbial cells as “micro-factories,” leveraging their inherent enzyme systems and cofactor networks to provide a stable microenvironment, thereby significantly reducing costs and improving operational stability [12].

*Aspergillus oryzae* is a generally recognized as safe (GRAS) filamentous fungus with a long history of use in the food industry and considerable potential as a host for whole-cell biocatalysis [13], owing to its strong protein secretion capacity and well-developed metabolic pathways. In our previous study [14], we established that *A. oryzae* can efficiently catalyze gastrodin propionylation in ionic liquid-containing systems via whole-cell biocatalysis, with comprehensive characterization of the reaction products and optimization of process parameters. However, the mechanistic basis for the differences in catalytic efficiency observed under various induction conditions remained unexplored. Many microbial lipases are inducible, and the type and concentration of inducers significantly affect their activity and expression levels [15]. Studies have shown that the efficiency of whole-cell biocatalysts is highly dependent on the inducer type, with differences likely linked to the regulatory expression of specific intracellular lipases [16]. However, due to the complexity of whole-cell systems, conventional enzymological approaches are insufficient to systematically reveal the global regulatory effects of inducers on the metabolic network and key enzyme gene expression in *A. oryzae*, thus limiting the understanding of overall catalytic performance.

Transcriptomics, as a high-throughput technology, enables genome-wide profiling of gene expression differences, providing a powerful tool for systematically deciphering microbial metabolic regulation mechanisms [17]. By comparing gene expression profiles under different culture conditions (e.g., different inducers), key differentially expressed genes (DEGs) involved in specific metabolic pathways can be precisely identified, thereby explaining phenotypic differences at the molecular level [18,19]. In recent years, this approach has been successfully applied to elucidate microbial stress responses, secondary metabolite biosynthesis, and enzyme induction mechanisms [20].

Based on this background, this study employed comparative transcriptomics to analyze the induction regulation mechanism of lipases in *A. oryzae* whole cells at the transcriptional level, focusing on the expression patterns of lipases related to gastrodin propionylation. This research is significant for clarifying the localization, expression, and regulation of whole-cell lipases, not only providing molecular insights into the inducible expression regulation of *A. oryzae* lipases but also establishing a theoretical foundation for the rational engineering of *A. oryzae* to construct high-efficiency whole-cell biocatalysts.

## 2. Materials and Methods

### 2.1. Microorganism and Chemicals

*Aspergillus oryzae* GIM 3.482 was obtained from the Guangdong Culture Collection Center (Guangzhou, China). Gastrodin (97% purity) and vinyl propionate (VP, 98% purity) were supplied by TCI (Tokyo, Japan). 1-Hexyl-3-methylimidazolium hexafluorophosphate ([HMIM][PF6], 97% purity) was purchased from Aladdin Industrial Corporation (Shanghai, China). All other chemicals were acquired from Macklin Inc. (Shanghai, China).

### 2.2. Cultivation and Induction of A. oryzae

*A. oryzae* spores were initially activated on potato dextrose agar (PDA) medium at 28 °C for 60 h. A spore suspension was prepared by harvesting spores with sterile distilled water. This suspension (3%, *v*/*v*) was inoculated into a liquid medium and cultivated at 37 °C with shaking at 180 rpm for 48 h.

The liquid medium consisted of 5 g/L yeast extract, 5 g/L (NH_4_)_2_SO_4_, 1 g/L KH_2_PO_4_, and 0.2 g/L MgSO_4_·7H_2_O. After sterilization, a filter-sterilized carbon source/inducer was added to a final concentration of 5.0 g/L. Different inducer groups were established, including glucose, Tween-80, Triton X-100, oleic acid, olive oil, and soybean oil.

After cultivation, mycelia were harvested by vacuum filtration, washed thoroughly with distilled water to remove residual medium, and then freeze-dried under vacuum, and stored at −80 °C. The freeze-dried cells were then rehydrated and used for biocatalysis for future use.

### 2.3. Propionylation of Gastrodin by A. oryzae Whole Cells in Ionic Liquid-Containing System

The propionylation reaction catalyzed by *A. oryzae* whole cells was performed according to our previously published method [14]. Briefly, a pyridine-[HMIM][PF_6_] (50/50, *v*/*v*) biphasic solvent system was established. The reaction was conducted in 1 mL of this solvent system containing gastrodin (20 mM), *A. oryzae* whole-cell biocatalyst (40 mg/mL, dry weight), and vinyl propionate (600 mM) at 40 °C with agitation at 180 rpm. Whole-cell catalysts were prepared from *A. oryzae* mycelia induced with different inducers. The conversion rate of gastrodin propionylation and the biomass yield were determined.

### 2.4. Detection and Quantification of Substrate and Products

The detection and quantification of gastrodin and its propionylated derivatives were performed using reversed-phase high-performance liquid chromatography (RP-HPLC). The analytical method was established and validated in our previous study [14], with minor modifications for the current work.

Chromatographic analysis was conducted using a Waters HPLC system equipped with a Zorbax SB-C18 column (4.6 mm × 250 mm, 5 μm; Agilent Technologies, Santa Clara, CA 95051, USA) equipped with a Waters 600E pump and a Waters 2475 fluorescence detector (Waters Corp., Milford, MA 01757, USA). Detection wavelengths were set at an excitation of 268 nm and an emission of 290 nm. A mobile phase of methanol and water (40:60, *v*/*v*) was used at a flow rate of 0.9 mL min^−1^.

The conversion rate was calculated using the following formula:

$$Conversion\;\left(\%\right)=\frac{\left(S0-St\right)}{S0}\times 100\%$$

where S0 represents the peak area of gastrodin before the reaction, and St represents the peak area after reaction time t hours.

The identity of the propionylated product (gastrodin propionate) was confirmed by comparison with authentic standards and LC-MS analysis as described in previous publication, ensuring the specificity of the analytical method.

### 2.5. Biomass Quantification

For biomass determination, mycelia were harvested by vacuum filtration, washed thoroughly to remove residual medium, and then freeze-dried for 48 h under vacuum. The resulting dried mycelial powder was weighed to determine the biomass yield under each cultivation condition.

All analytical measurements were performed in triplicate to ensure reproducibility, and results are presented as mean ± standard deviation.

### 2.6. RNA Library Construction and Transcriptome Sequencing

Mycelia from target cultures were rapidly collected by vacuum filtration under cold conditions, washed three times with PBS buffer to remove surface medium, immediately frozen in liquid nitrogen, and stored at −80 °C for RNA extraction.

Total RNA was extracted from the thawed mycelia using the Trizol method. RNA quality was assessed, and samples meeting quality standards were used for library construction. Libraries were prepared following the instructions of the DNBSEQ Library Prep Kit (MGI Tech Co., Ltd., Shenzhen, China) and sequenced on the MGISEQ-2000 platform.

Raw sequencing data were filtered using SOAPnuke. Clean reads were aligned to the reference genome using Bowtie2, and gene/transcript expression levels were quantified with RSEM. De novo assembly of clean reads was performed using Trinity, and the assembled transcripts were clustered to remove redundancy using Tgicl, resulting in a set of Unigenes.

Gene expression levels were calculated using the FPKM method. Differentially expressed genes (DEGs) were identified using the DESeq2 R package 1.50.2 with the criteria of |log_2_(fold change)| > 1 and adjusted *p*-value < 0.05, with the glucose-induced group as the control and the soybean oil-induced group as the experimental condition.

Unigenes were annotated by BLAST 2.12.0 alignment against public databases including NCBI NR/NT databases. Functional enrichment analyses were conducted using the Gene Ontology (GO) and Kyoto Encyclopedia of Genes and Genomes (KEGG) databases to identify key pathways and gene sets involved in the cellular response to different inducers.

### 2.7. Homology Modeling and Molecular Docking

The three-dimensional structure of the target gene product was modeled using SWISS-MODEL. Templates with sequence similarity greater than 30% were selected for homology modeling based on the target enzyme’s amino acid sequence. The resulting models were evaluated, and a high-scoring lipase model was selected for further study. Molecular docking of the enzyme with gastrodin was performed using AutoDock MGL Tools 1.5.6 to investigate substrate-enzyme interactions. The docking results were visualized using PyMOL 3.1.3.

### 2.8. Statistical Analysis

All experiments were performed in at least triplicate, and data are presented as mean ± standard deviation (SD). Data analysis was conducted using OriginPro 2024 (OriginLab Corporation, Northampton, MA, USA). Statistical significance was determined by one-way analysis of variance (ANOVA) followed by Duncan’s test, with a *p*-value < 0.05 considered statistically significant.

## 3. Results and Discussion

### 3.1. Effect of Inducer Type on the Catalytic Activity of A. oryzae Whole Cells in Gastrodin Propionylation and on Biomass

The propionylation of gastrodin using *A. oryzae* whole-cell biocatalysis in ionic liquid-containing systems was established in our previous work [14], where it comprehensively validated the reaction system, including product identification and quantification methods. Building upon this established protocol, the current study investigated the differential catalytic efficiency under various induction conditions.

**Figure 1:**
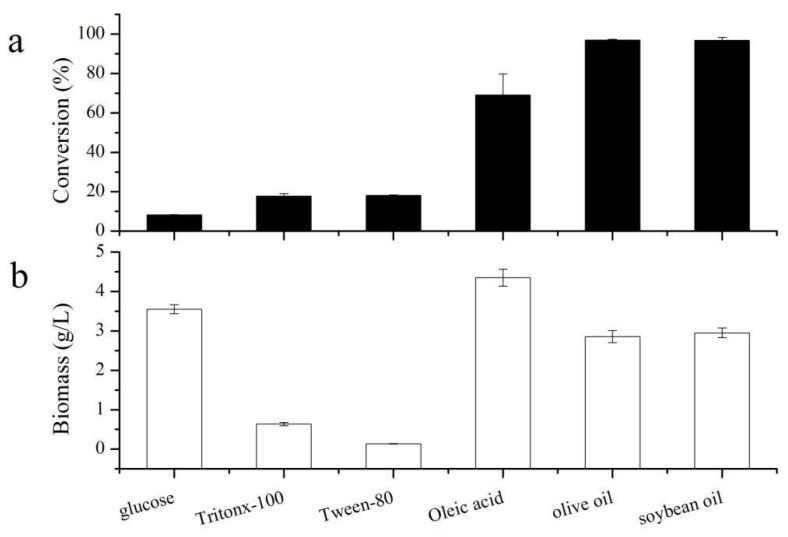
The influence of different inducers on (**a**) the conversion rate of gastrodin propionylation and (**b**) the biomass of *Aspergillus oryzae*.

Many microbial lipases are inducible enzymes, whose activity and quantity are influenced by the type and concentration of the inducer [21]. As shown in Figure 1a, when olive oil was used as the inducer, the conversion rate of gastrodin propionylation reached the highest value of 96.99%, similar to the activity observed with soybean oil induction (96.84%). This was followed by oleic acid induction, yielding a conversion rate of 69.04%. The lowest conversion rate (8.23%) was obtained with glucose induction. The high conversion rate (>96%) and selectivity observed suggest that the isolated yield of gastrodin propionate would closely approach the conversion efficiency.

Regarding biomass (Figure 1b), cultures induced with glucose, oleic acid, olive oil, and soybean oil all yielded high biomass, exceeding 2.85 g/L. The highest biomass (4.35 g/L) was observed with oleic acid induction. In contrast, biomass was generally low (<1.00 g/L) when surfactants Tween-80 and Triton X-100 were used as inducers. Comparing the relationship between biomass and conversion rate revealed that high biomass did not necessarily correlate with high catalytic activity, underscoring the necessity of investigating the effect of inducer type on the catalytic performance of *A. oryzae*. Oils and fats, including corn oil, soybean oil, olive oil, as well as surfactants like Tween-80, are known to be effective inducers for lipase production, a phenomenon observed in various lipase-producing microorganisms [22,23,24]. The performance of glucose as an inducer varies among strains; its poor performance in this study might be attributed to carbon catabolite repression (CCR) in *A. oryzae* [25].

Although both soybean oil and olive oil yielded high conversion rates, soybean oil was selected for further transcriptomic analysis based on its cost-effectiveness. Based on the inducer performance, the glucose-induced group and the soybean oil-induced group were selected as representative conditions for further transcriptomic analysis to elucidate the molecular mechanisms underlying the inducer-dependent catalytic efficiency.

### 3.2. RNA Quality Assessment and De Novo Assembly Evaluation

RNA quality assessment and the evaluation of de novo assembly results are critical steps for ensuring the reliability, accuracy, and reproducibility of the research findings. As shown in Table 1, the RNA extracted from *A. oryzae* met the required quality standards, showing high purity, integrity, and minimal degradation, indicating high reliability of the constructed libraries. The statistics of the de novo transcriptome assembly are presented in Table 2. A total of 47,718 Unigenes were assembled from all *A. oryzae* samples, with a high N50 value (5559 bp) and a mean unigene length of 3762 bp for the combined assembly, indicating high assembly completeness and continuity, which ensures reliability for subsequent differential gene expression analysis.

### 3.3. Global Gene Expression and Identification of DEGs

The assembled Unigenes were aligned against various databases for functional annotation. The results, summarized in Table 3, showed that 44,142 Unigenes (92.51%) were annotated in the NR database. A total of 28,719 Unigenes were annotated in the GO database, distributed across biological processes (15,199), molecular functions (22,607), and cellular components (17,653). Furthermore, 35,860 Unigenes were annotated to the KEGG database and mapped to 126 KEGG pathways. These annotations facilitated the investigation of metabolic regulation mechanisms in *A. oryzae* in response to different inducers.

**Figure 2:**
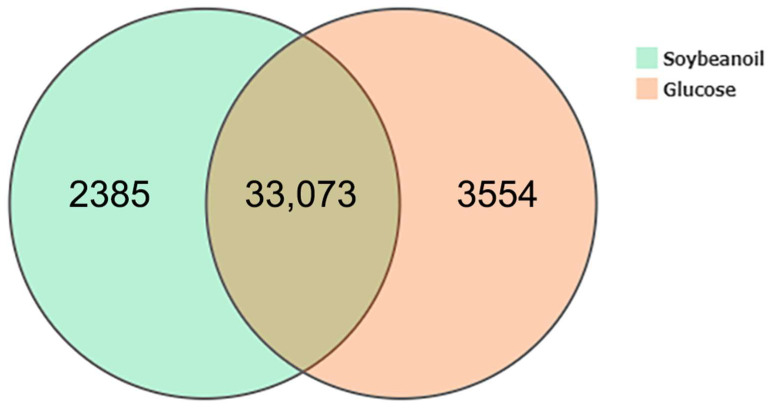
Venn diagram comparing gene expression profiles between the glucose-induced and soybean oil-induced groups.

A Venn diagram comparing gene expression between the two groups (Figure 2) revealed that 33,073 genes were co-expressed, representing the core set of genes required for basic cellular activities. Notably, 2385 genes (6.7%) were specifically expressed in the soybean oil group, while 3554 genes (9.7%) were specific to the glucose group. This indicates that the type of inducer significantly and globally alters the gene expression profile of *A. oryzae*, reflecting specific regulatory adaptations to different carbon source environments.

**Figure 3:**
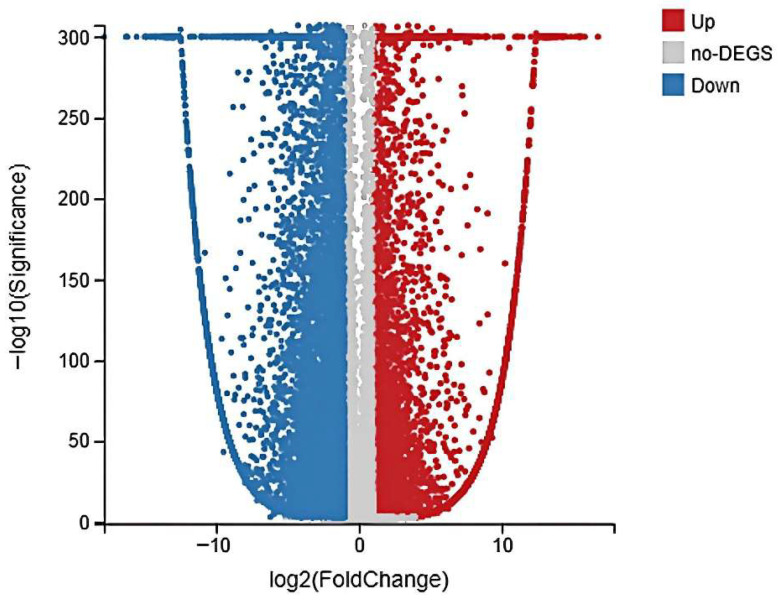
Volcano plot of differentially expressed genes (DEGs) between soybean oil-induced and glucose-induced *Aspergillus oryzae*. Red dots represent significantly upregulated genes, blue dots represent significantly downregulated genes, and gray dots represent non-DEGs.

DEGs between the soybean oil group and the glucose group were identified using stringent criteria (Q-value < 0.001 and |Log_2_ FC| > 1) to ensure reliability. As shown in the volcano plot (Figure 3), a total of 20,342 DEGs were identified, comprising 6514 significantly upregulated genes and 13,828 significantly downregulated genes in the soybean oil group compared to the glucose group. Functional annotation of these DEGs was performed to elucidate the impact of the inducer

### 3.4. GO Functional Enrichment Analysis of DEGs

**Figure 4:**
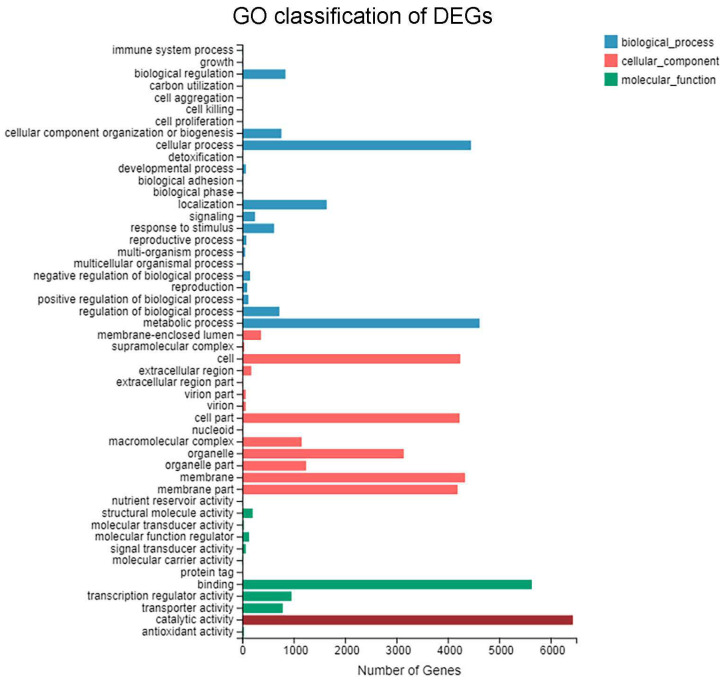
GO classification of DEGs between the soybean oil-induced and glucose-induced groups.

GO functional analysis was performed to systematically categorize the DEGs, revealing their potential involvement in biological processes, cellular components, and molecular functions. The results (Figure 4) showed that 61.5% (12,521) of the DEGs were annotated to the GO database, indicating comprehensive coverage. These DEGs were assigned to 2256 GO terms. The ‘cellular component’ category contained the highest number of terms, followed by ‘biological process’, while ‘molecular function’ contained the fewest. Within ‘biological process’, the subcategories ‘metabolic process’ and ‘cellular process’ were the most highly enriched. Under ‘cellular component’, the subcategories ‘cell’, ‘cell part’, ‘membrane’, and ‘membrane part’ contained the largest number of DEGs. For ‘molecular function’, the subcategories ‘catalytic activity’ and ‘binding’ were the most significantly enriched. These results demonstrate, at the gene expression level, that induction with different carbon sources significantly affects the expression of genes related to catalytic functions in *A. oryzae*.

### 3.5. KEGG Pathway Enrichment Analysis of DEGs

**Figure 5:**
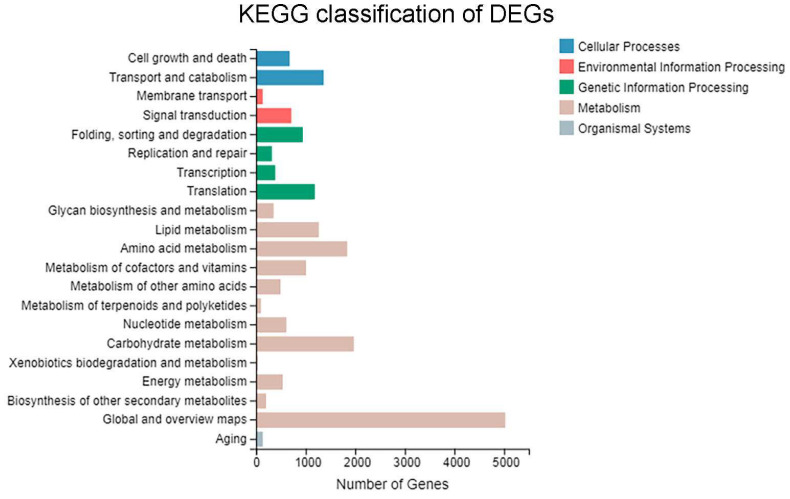
KEGG pathway classification of DEGs between samples.

KEGG pathway analysis was conducted to identify metabolic and signaling pathways significantly enriched with DEGs, thereby linking transcriptomic changes to potential cellular functional mechanisms. The classification analysis (Figure 5) indicated that 9671 DEGs were significantly enriched in 126 KEGG pathways, which were broadly categorized into five major groups: Metabolism, Genetic Information Processing, Cellular Processes, Environmental Information Processing, and Organismal Systems. The ‘Metabolism’ category contained the largest number of entries, followed by ‘Genetic Information Processing’.

**Figure 6:**
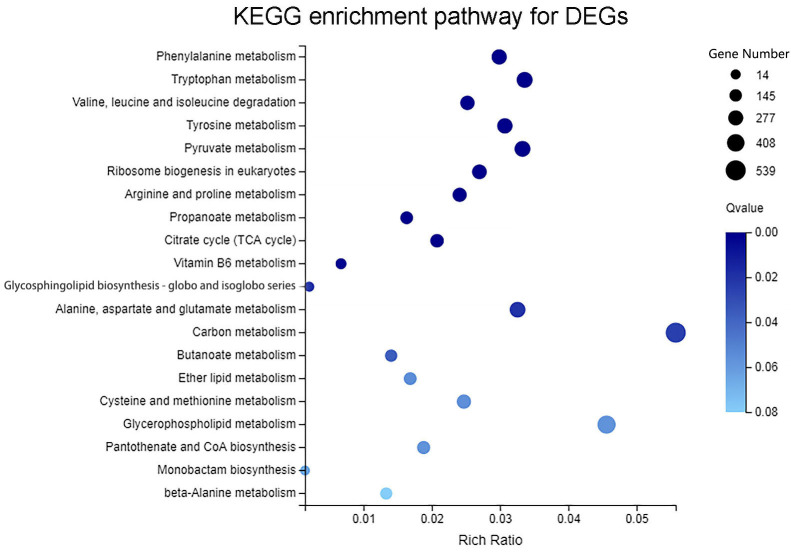
Scatter plot of KEGG pathway enrichment analysis for DEGs between the soybean oil-induced and glucose-induced groups. The rich factor represents the degree of enrichment. The size of the dots corresponds to the number of DEGs in that pathway, and the color indicates the range of the Q-value.

Further KEGG pathway enrichment analysis revealed that the most significantly enriched metabolic pathways for the DEGs were Carbon metabolism, Glycerophospholipid metabolism, Tryptophan metabolism, and Pyruvate metabolism (Figure 6). These results indicate that treatment with soybean oil versus glucose caused significant differences in gene expression primarily related to metabolic pathways and genetic information processing, particularly involving carbohydrate metabolism, amino acid metabolism, and lipid metabolism.

### 3.6. Prioritization of Key Candidate Lipase Genes from DEGs

The dramatic difference in catalytic conversion between the glucose- and soybean oil-induced groups (8.23% vs. 96.84%) strongly suggested a fundamental shift in the lipase expression profile of *A. oryzae*.

To identify the most plausible candidate responsible for gastrodin propionylation, a stepwise filtering strategy was employed on the 26 upregulated lipase-related DEGs (Table 4). These included genes annotated as lysophospholipase [EC:3.1.1.5] (2 genes), triacylglycerol lipase [EC:3.1.1.3] (4 genes), acetylcholinesterase [EC:3.1.1.7] (2 genes), acylgycerol lipase [EC:3.1.1.23] (1 gene), phospholipase C [EC:3.1.4.3] (5 genes), phospholipase D1/2 [EC:3.1.4.4] (9 genes), and phosphatidylinositol phospholipase C, delta [EC:3.1.4.11] (3 genes).

First, genes encoding enzymes with high substrate specificity for phospholipids (e.g., lysophospholipases, phospholipases C and D) were excluded from consideration, as their native functions are less likely to involve the acylation of a phenolic glycoside like gastrodin. This rationale focused subsequent analysis on enzymes with broader substrate specificity, primarily the four triacylglycerol lipases [EC:3.1.1.3] (Table 5), as they are reported commonly involved in acylation reactions.

### 3.7. Homology Modeling and Molecular Docking of the Differentially Expressed Lipase

Molecular docking serves as a valuable computational tool for predicting substrate-enzyme interactions, particularly when coupled with transcriptional evidence of enzyme induction. While transcriptomics revealed the induction patterns of lipase genes, molecular docking provided mechanistic insights into their catalytic competence. Homology modeling was attempted for the shortlisted triacylglycerol lipase genes (Table 5). The final prioritization was based on structural competence.

Analysis of the SWISS-MODEL results indicated that only the amino acid sequence derived from gene CL24.Contig40_All could form a complete and valid lipase structure. This was likely because the de novo RNA assembly yielded incomplete sequences for the other candidates; CL24.Contig67_All and Unigene4417_All appeared to be partial gene fragments insufficient for forming a functional enzyme structure. Furthermore, no amino acid sequence was available from the transcriptome data for CL24.Contig17_All, preventing its analysis.

**Figure 7:**
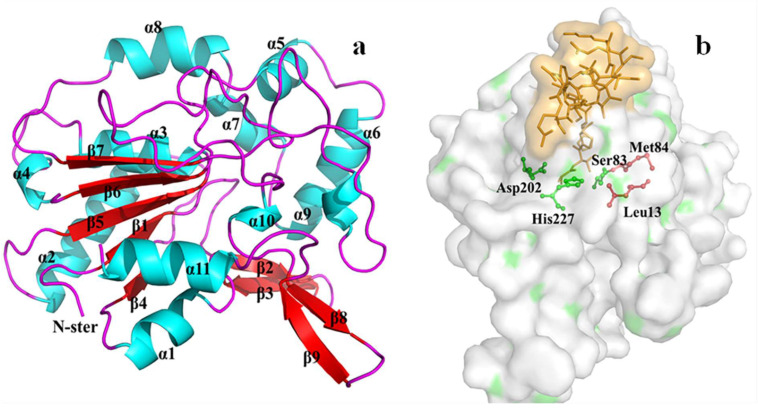
Homology modeling of Lipase CL24_40. (**a**) Overall structure of the enzyme showing the α/β hydrolase fold. (**b**) Detailed view of the catalytic core. The catalytic triad (Ser83, Asp202, His227) is shown in green. Residues forming the oxyanion hole (Leu13, Met84) are shown in red. The lid domain is colored gold.

The successfully modeled enzyme based on CL24.Contig40_All was designated Lipase CL24_40. Structural analysis revealed that Lipase CL24_40 possesses a canonical α/β hydrolase fold, consisting of 9 β-strands and 11 α-helices connected by loops (Figure 7a). The catalytic triad, essential for hydrolysis, was identified as Ser83, Asp202, and His227 (Figure 7b).

A “lid” domain, typically involved in interfacial activation, was identified as the region Ala117-Glu129. This lid forms an amphipathic α-helix, where hydrophobic residues (Ala117, Val118, Ala119, Ile122, Leu123) face the enzyme’s interior and hydrophilic residues (Asp120, Tyr121, Lys124) are solvent-exposed. This structure is characteristic of lipases exhibiting interfacial activation, a phenomenon where enzymatic activity sharply increases at the oil-water interface when the substrate concentration exceeds its critical micelle concentration (CMC). Activation involves a conformational change where the lid opens, exposing the hydrophobic active site [26,27]. The oxyanion hole, crucial for stabilizing the transition state during catalysis, was formed by the NH groups of Leu13 and Met84 (Figure 7b).

**Figure 8:**
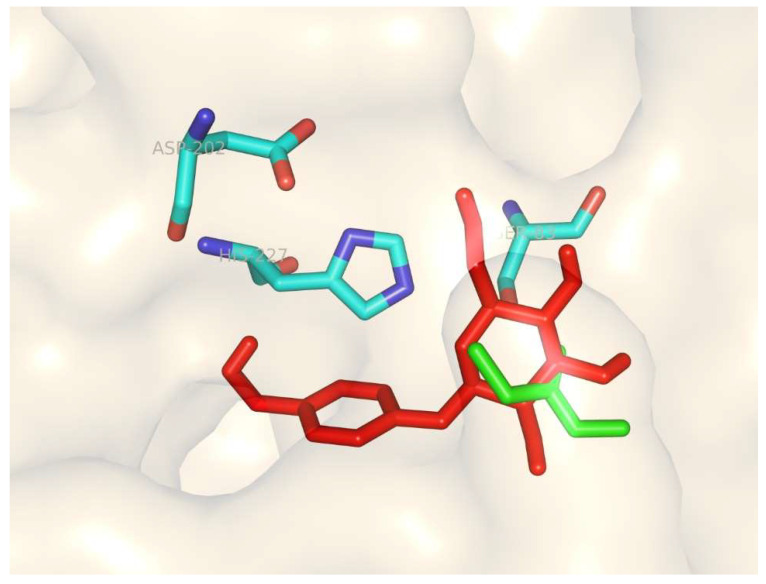
Molecular docking model of Lipase CL24_40 with gastrodin and VP. Gastrodin is represented by red sticks, and VP is represented by green sticks, positioned within the enzyme’s active site.

Molecular docking studies were performed using the Lipase CL24_40 model (Figure 8). Based on the general catalytic mechanism of lipases [28], the propionylation reaction was inferred to proceed as follows: VP binds to the active site. His227 deprotonates Ser83, enabling the nucleophilic oxygen of Ser83 to attack the carbonyl carbon of VP, forming a tetrahedral intermediate stabilized by the oxyanion hole. This intermediate collapses, releasing acetaldehyde and forming an acyl-enzyme intermediate. Subsequently, the deprotonated hydroxyl group of gastrodin attacks the acyl-enzyme intermediate, leading to the formation of gastrodin propionate and regeneration of the free enzyme.

### 3.8. Analysis of the Regulatory Mechanism Underlying High Lipase Expression Induced by Soybean Oil

Based on the transcriptomic profiles and the identification of upregulated lipases (Table 6), it can be proposed that the enhanced catalytic activity under soybean oil induction may be attributed to the following two interconnected mechanisms:

(1) Accelerated Lipid Metabolism: Key lipases involved in glyceride metabolism, namely triacylglycerol lipase [EC:3.1.1.3] and acylgycerol lipase [EC:3.1.1.23], were highly expressed in the soybean oil group. Triacylglycerol lipase catalyzes the hydrolysis of triacylglycerols to diacylglycerols and fatty acids, while acylgycerol lipase further hydrolyzes monoacylglycerols to glycerol and fatty acids. The coordinated upregulation of these enzymes indicates an accelerated lipid catabolism pathway in *A. oryzae* when induced with soybean oil, leading to increased lipase production.

(2) Signal Transduction Processes: Phosphatidylinositol-specific phospholipase C (PLC) was found to be highly expressed in the soybean oil-induced group. This enzyme hydrolyzes phosphatidylinositol 4,5-bisphosphate (PIP2) to generate diacylglycerol (DAG) and inositol 1,4,5-trisphosphate (IP3), both of which are important secondary messengers [29]. DAG activates protein kinase C (PKC), which in turn phosphorylates target proteins and regulates various cellular processes, including gene transcription [30,31]. Previous studies have indicated that the PLC-DAG-IP3-PKC signaling axis plays a crucial role in the regulation of lipid metabolism, a process also documented in yeast.

## 4. Conclusions

This study elucidates the molecular basis for the significantly higher efficiency of *Aspergillus oryzae* whole-cell biocatalyst in gastrodin propionylation when induced by soybean oil versus glucose. Comparative transcriptomics revealed extensive metabolic reprogramming, with 20,342 DEGs enriched in lipid metabolic and phosphatidylinositol signaling pathways. Among 26 upregulated lipase-related genes, a candidate triacylglycerol lipase (CL24.Contig40_All) was prioritized through multi-step bioinformatic screening. Homology modeling confirmed its typical α/β hydrolase fold with a functional catalytic triad, and molecular docking demonstrated favorable binding with both gastrodin and vinyl propionate. These findings indicate that soybean oil induction enhances lipase expression by activating lipid catabolism and PLC-mediated signaling, providing crucial transcriptional insights and a genetic target for designing efficient whole-cell biocatalysts for natural product modification.

Compared to prior transcriptomic studies on lipase gene expression, this work not only identifies key differentially expressed lipases but also integrates homology modeling and molecular docking to prioritize a candidate gene, providing a multi-level mechanistic understanding of inducer-dependent efficiency. This work provides mechanistic insights into how soybean oil, as a complex natural inducer, activates both metabolic pathways and signaling cascades to enhance lipase expression, offering a more comprehensive understanding than studies using single chemical inducers. Finally, the identification of CL24.Contig40_All as a key candidate provides a specific genetic target for future metabolic engineering efforts, whereas many transcriptomic studies stop at pathway-level analysis without proposing concrete engineering strategies. As computational evidence cannot definitively establish functional relationships, future work should include experimental validation through gene knockout/complementation studies or heterologous expression and enzymatic characterization.

## Figures and Tables

**Table 1:** RNA quality assessment of the glucose-induced group (G) and soybean oil-induced (S) group of *Aspergillus oryzae*.

Sample Name	Concentration (ng/μL)	Volume (μL)	Amount (μg)	OD260/280	OD260/230	RIN	28S/18S
S_1	730	40	29.2	2.14	2.38	7.1	1.7
S_2	2863	40	114.52	0.98	1.12	5.7	2.2
S_3	1617	40	64.68	1.99	2.27	6.8	1.9
G_1	2632	40	105.28	1.45	1.66	6.6	3.1
G_2	2177	40	87.08	1.82	2.08	6.5	2.3
G_3	3024	20	60.48	1.51	1.74	6.8	1.8

Note: S_1, S_2, S_3: biological replicates of the soybean oil-induced group; G_1, G_2, G_3: biological replicates of the glucose-induced group.

**Table 2:** Statistics and quality assessment of the de novo transcriptome assembly for all samples.

Sample	Total Number	Total Length (bp)	Mean Length (bp)	N50 (bp)	N70 (bp)	N90 (bp)	GC(%)
G_1	33,926	78,394,507	2310	3534	2457	1302	49.59
G_2	40,428	115,325,163	2852	4463	3062	1642	49.52
G_3	31,948	70,971,079	2221	3231	2276	1280	49.67
S_1	23,499	43,553,160	1853	2818	1931	1018	49.93
S_2	33,692	99,384,502	2949	5255	3217	1529	49.38
S_3	22,370	40,272,927	1800	2790	1912	970	49.96
All-Unigene	47,718	179,560,378	3762	5559	3804	2067	49.42

Note: S_1, S_2, S_3: biological replicates of the soybean oil-induced group; G_1, G_2, G_3: biological replicates of the glucose-induced group.

**Table 3:** Number and ratio of annotated Unigenes of *Aspergillus oryzae*.

Database	Number of Annotated Unigenes	Annotation Ratio (%)
NR	44,142	92.51
NT	47,190	98.89
Swissprot	34,166	71.60
KEGG	35,860	75.15
KOG	30,809	64.56
Pfam	37,455	78.49
GO	28,719	60.18

**Table 4:** Differentially expressed genes related to lipase.

Gene ID	NR Annotation
CL117.Contig30_All	unnamed protein product [*Aspergillus oryzae* RIB40]
CL117.Contig6_All	unnamed protein product [*Aspergillus oryzae* RIB40]
CL117.Contig36_All	PLD-like domain protein [*Aspergillus parasiticus* SU-1]
CL117.Contig54_All	PLD-like domain protein [*Aspergillus parasiticus* SU-1]
CL117.Contig7_All	HLH transcription factor (GlcD gamma), putative [*Aspergillus oryzae* 3.042]
CL117.Contig12_All	phospholipase PldA [*Aspergillus oryzae* RIB40]
CL117.Contig50_All	phospholipase PldA [*Aspergillus oryzae* RIB40]
CL2062.Contig2_All	phospholipase D1 [*Aspergillus oryzae* 3.042] > KDE78745.1 phospholipase D1 [*Aspergillus oryzae* 100-8]
CL2062.Contig8_All	phospholipase D1 [*Aspergillus oryzae* 3.042] > KDE78745.1 phospholipase D1 [*Aspergillus oryzae* 100-8]
CL4019.Contig2_All	lysophospholipase 1 [*Aspergillus flavus* AF70]
CL2271.Contig7_All	hypothetical protein AN7792.2 [*Aspergillus nidulans* FGSC A4]
CL24.Contig17_All	triacylglycerol lipase [*Aspergillus bombycis*]
CL24.Contig40_All	triacylglycerol lipase [*Aspergillus oryzae* RIB40]
CL24.Contig67_All	triacylglycerol lipase [*Aspergillus bombycis*]
Unigene4417_All	lipase atg15 [*Aspergillus oryzae* RIB40]
CL542.Contig1_All	lipase/esterase, putative [*Aspergillus flavus* NRRL3357]
CL542.Contig4_All	lipase/esterase, putative [*Aspergillus flavus* NRRL3357]
CL726.Contig10_All	putative non-hemolytic phospholipase C precursor [*Aspergillus flavus* AF70]
CL726.Contig9_All	non-hemolytic phospholipase C precursor [*Aspergillus oryzae* RIB40]
CL726.Contig1_All	phospholipase C [*Aspergillus oryzae* 3.042] > KDE81680.1 phospholipase C [*Aspergillus oryzae* 100-8]
CL972.Contig11_All	phospholipase C [*Aspergillus oryzae* 3.042]
CL972.Contig7_All	phospholipase C [*Aspergillus oryzae* 3.042]
CL195.Contig2_All	alpha/beta hydrolase, putative [*Aspergillus flavus* NRRL3357]
CL31.Contig14_All	phosphoinositide-specific phospholipase C, putative [*Aspergillus oryzae* 3.042]
CL31.Contig7_All	phosphoinositide-specific phospholipase C, putative [*Aspergillus oryzae* 3.042]
CL31.Contig8_All	phosphoinositide-specific phospholipase C, putative [*Aspergillus oryzae* 3.042]

**Table 5:** DEGs annotate as triacylglycerol lipase.

Gene ID	NR Annotation
CL24.Contig17_All	triacylglycerol lipase [*Aspergillus bombycis*]
CL24.Contig40_All	triacylglycerol lipase [*Aspergillus oryzae* RIB40]
CL24.Contig67_All	triacylglycerol lipase [*Aspergillus bombycis*]
Unigene4417_All	lipase atg15 [*Aspergillus oryzae* RIB40]

**Table 6:** Metabolic pathways involving the differentially expressed lipases.

Pathway Name	Pathway ID	Term Candidate Gene Num
Glycerophospholipid metabolism	ko00564	18
Ether lipid metabolism	ko00565	14
Inositol phosphate metabolism	ko00562	8
Endocytosis	ko04144	9
Glycerolipid metabolism	ko00561	5
Phosphatidylinositol signaling system	ko04070	3

## Data Availability

The original contributions presented in this study are included in the article. Further inquiries can be directed to the corresponding author(s).
